# Swirl-like Acoustofluidic Stirring Facilitates Microscale Reactions in Sessile Droplets

**DOI:** 10.3390/mi14040837

**Published:** 2023-04-12

**Authors:** Huaize Lan, Jingui Qian, Yansong Liu, Shanshan Lu, Bowei Zhang, Liang Huang, Xuefeng Hu, Wei Zhang

**Affiliations:** 1 Anhui Province Key Laboratory of Measuring Theory and Precision Instrument, School of Instrument Science and Opto-Electronics Engineering, Hefei University of Technology, Hefei 230009, Chinazhangw@hfut.edu.cn (W.Z.); 2 Special Display and Imaging Technology Innovation Center of Anhui Province, Academy of Optoelectronic Technology, Hefei University of Technology, Hefei 230009, China

**Keywords:** SAWs, sessile droplets, acoustic swirls, acoustic microdevices, stirring

## Abstract

Sessile droplets play a crucial role in the microreactors of biochemical samples. Acoustofluidics provide a non-contact and label-free method for manipulating particles, cells, and chemical analytes in droplets. In the present study, we propose a micro-stirring application based on acoustic swirls in sessile droplets. The acoustic swirls are formed inside the droplets by asymmetric coupling of surface acoustic waves (SAWs). With the merits of the slanted design of the interdigital electrode, the excitation position of SAWs is selective by sweeping in wide frequency ranges, allowing for the droplet position to be customized within the aperture region. We verify the reasonable existence of acoustic swirls in sessile droplets by a combination of simulations and experiments. The different periphery of the droplet meeting with SAWs will produce acoustic streaming phenomena with different intensities. The experiments demonstrate that acoustic swirls formed after SAWs encountering droplet boundaries will be more obvious. The acoustic swirls have strong stirring abilities to rapidly dissolve the yeast cell powder granules. Therefore, acoustic swirls are expected to be an effective means for rapid stirring of biomolecules and chemicals, providing a new approach to micro-stirring in biomedicine and chemistry.

## 1. Introduction

Recently, droplet microfluidics have been regarded as one of the most powerful tools in the field of analytical chemistry and biomedicine, such as reagent delivery [1] and drug encapsulation [2]. The main types of manipulation of micro-sized organisms in microfluidics are optical tweezers, magnetic tweezers [3], and acoustic tweezers [4]. Optical tweezers use intensity high-intensity light to capture organisms [5], which requires high energy and reduces cell viability. Magnetic tweezers capture organisms by magnetic force, which requires pre-treatment of the organism [6], limiting its application in biochemistry. Acoustic tweezers based on surface acoustic waves (SAWs) allow for low-power, harmless and non-contact manipulation [7]. SAW devices have the advantages of real-time manipulation, programmability [8], label-free detection capability [9], and miniaturization. Therefore, SAW devices have unique advantages compared to optical and magnetic tweezers. 

**Figure 1:**
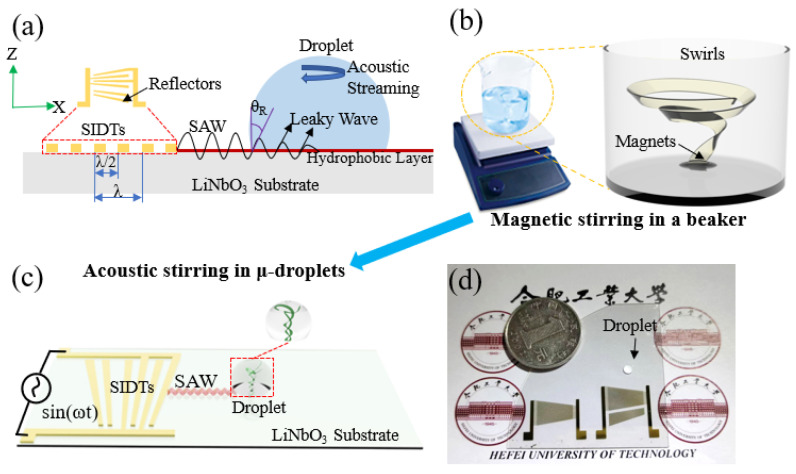
(**a**) Working principle of SAWs propagation and the generation of acoustic streaming. (**b**,**c**) Schematic diagram of acoustolfuidic μ-droplets stirring method for updating magnetic stirring in a beaker. (**d**) Optical image of the fabricated acoustic microdevices.

As far as current developments are concerned, microchannels and open droplet platforms are two important branches of acoustofluidics. The acoustic microchannel systems consisting of microchannel, pipes, and syringe pumps have the disadvantages of being complex and not portable [10], which can greatly increase the operational difficulty and time cost [11]. The droplet-based platform is a simpler and more flexible system compared with the microchannel platform, which avoids the complex and difficult fabrication process, thus achieving reuse [12]. A sessile droplet or another fluid is added directly dropwise to the piezoelectric substrate, and then SAWs are scattered as asymmetric waves to the interior of the droplet. The SAW action on droplets is attributed to two forces: the drag force caused by the acoustic radiation force (ARF) and the acoustic streaming force (ASF) [13], which can induce acoustic streaming. The acoustic streaming formation is based on asymmetric SAWs coupling into the droplet interior. As the propagation velocity of sound in solids is greater than that in liquids, SAWs are converted into leakage wave modes and propagate in droplets in the form of Rayleigh angles [14] at the moment of meeting the droplet (see Figure 1a). Different acoustic streaming phenomena can manipulate particles or cells inside the droplet in different ways, such as the formation of particle rings [15], particle enrichment [16,17,18], particle alignment [19], cell lysis [20], sample mixing [21]. patternable manipulation technique [22].

Various acoustic steaming phenomena have been widely applied, but the applications of acoustic swirls have yet to be explored in depth. J. L. Han et al. used high-frequency standing SAWs to induce acoustic swirl to separate particles [23]. Alghane, M. et al. analyzed the acoustic swirls by numerical analysis and experiments [24], only considering the acoustic swirls as an acoustic phenomenon; however, the application of acoustic swirls in micro-stirring was not discussed in detail. Magnetic stirring is used by many biochemical laboratories (see Figure 1b), but magnetic stirring not only requires a large amount of sample, and its stirring efficiency is not high, which is especially not suitable for expensive and rare sample stirring. Therefore, a micromixer capable of stirring micro-samples is extremely valuable; the acoustic swirls-based acoustic microdevices mentioned in this paper can accomplish the challenge of micro-stirring (see Figure 1c).

Conventional interdigital transducers (IDTs) only excite single-frequency SAWs with a wide and non-adjustable frequency and require a filter such as a phononic crystal [25,26] or droplet placed on the edge of IDTs [27] to generate asymmetric SAWs. To simplify the device structure, we used slanted interdigital transducers (SIDTs) to form a narrow beam of SAWs [28,29] in order to induce acoustic swirls. The numerical analysis of SAW propagation and the acoustic pressure gradient inside the droplet was performed using COMSOL Multiphysics 6.0, demonstrating that the acoustic swirls observed in the experiment are consistent with the simulation results of the acoustic pressure gradient formed by the acoustic wave propagation in droplets at a specific Rayleigh angle. SAWs cause temperature changes during transmission and capture the size and propagation trajectory of the narrow wave beam excited by SIDTs using a thermal imager.

By cleverly adding an oil phase environment between the SIDTs and the droplet, the resonant frequency required to induce acoustic swirls can be quickly determined by matching the direction of the oil beads rolling excited by the SAWs with the acoustic streaming phenomenon inside the droplet using the frequency sweeping. In the present study, the yeast cell powder granules were successfully dissolved and stirred in an 8 μL droplet by the acoustic swirls. The acoustic swirls based on droplets demonstrate excellent potential for micro stirring, and they are expected to accelerate the research process from large-size magnetic stirring to micro stirring in biology, chemistry, and medicine, realizing a real sense of “lab on a chip”.

## 2. Materials and Methods

### 2.1. Acoustic Microdevices Fabrication

To fabricate the acoustic microdevices, 20 pairs of SIDTs and 10 pairs of reflectors were designed, where the presence of the reflectors enhanced the unidirectional propagation of acoustic energy, all with widths from 50 μm to 100 μm, corresponding to SAWs wavelengths (*λ*) from 200 μm to 400 μm and resonant frequencies (*f*) from 10 MHz to 20 MHz (*f* = *Cs/λ*, *Cs* = 3950 ms^−1^ is the sound velocity of LiNbO_3_ (LN) piezoelectric substrate in the x-direction). The designed mask pattern was transferred on the surface of 128°Y-cut X-propagation LN by a standard photolithography process. A 5 nm Cr metal layer and a 50 nm Au metal layer were deposited on the LN surface using thermal evaporation. The electrodes were formed after the lift-off process by putting the LN substrate into acetone for several minutes. A Cr layer deposited between the LN and Au layers helped to improve the adhesion of Au. Figure 1d shows a photo of the fabricated acoustic microdevices.

### 2.2. Experimental Setup

The input signal of SIDTs was generated by the signal generator (DG4102, RIGOL, China) and amplified by the power amplifier (ZHL-5W-1+, Mini-Circuit, New York, NY, USA) in conjunction with the DC power supply (GPS-2303C, GWINSTEK, Suzhou, China). The actual working frequency of acoustic microdevices was detected by the vector network analyzer (T5260C, TRANSCOM INSTRUMENTS, Shanghai, China) as about 10 MHz~20 MHz. The droplet behavior was recorded using a video microscope (AO-HD206, AOSVI, Shenzhen, China) and was illuminated by an external light source for reducing the reflection.

### 2.3. Sample Preparation

In this work, green polystyrene (PS) particles with a diameter of 9 µm were diluted in deionized water to 2% (*v*/*v*). Yeast cell powder granules with a diameter of approximately 373 µm and a length of approximately 963 µm were used as a stirring demonstration experiment.

### 2.4. Numerical Simulation and Theoretical Analysis

**Figure 2:**
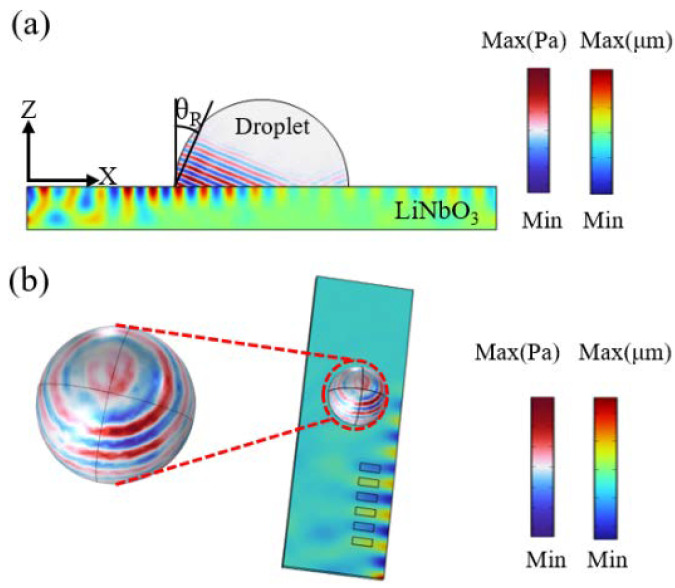
Simulation results of SAWs from the LN surface to scattering into the droplet interior using COMSOL Multiphysics 6.0. (**a**) 2D simulation results in SAWs propagating from left to right along the *X*-axis of the LN surface and scattering as the Rayleigh angle inside the droplet. *θ_R_* is the Rayleigh angle. (**b**) 3D simulation results of forming swirl-like acoustic pressure inside droplets by SAWs.

When SAWs met the droplet, they were scattered in the form of the Rayleigh angle, forming leakage waves, and the lost energy was converted into internal and mechanical energy of the fluid, which caused the fluid to form acoustic swirls and the remaining energy continues to propagate in the fluid in the mode of leakage waves. Two-dimensional (2D) finite element simulation expressed the mechanism of SAWs from generation to entering the fluid interior as shown in Figure 2a (see Video S1), and the Rayleigh angle $\theta_{R}\;$ is given by [30]

(1) $$\theta_{R}=\arcsin(\frac{C_{W}}{C_{S}})$$

where *C_W_* ≈ 1480 ms^−1^ is the speed of sound in fluid (water). 

The SAWs propagation in a sessile droplet on LN mainly depends on *αD* and Λ. The magnitude of force (*F*) is given by [31]

(2) $$F=f_{1}(\Lambda ,\alpha D)\frac{\rho_{0}\omega^{2}\mu_{0}{}^{2}\Lambda }{D}$$

where *f*_1_(Λ, *αD*), *α*, *D*, Λ, *ρ_0_*, *ω*, and *μ*_0_ are the geometrical distribution of the acoustic field, the attenuation rate, droplet diameter, a dimensionless parameter representing the transmission efficiency of Rayleigh wave to liquid, the density of the fluid, the harmonic frequency, and the magnitude of acoustic perturbation displacement, respectively [31]. We obtain scaling of the average fluid velocity (〈*V*〉) in droplets at moderate drive power

(3) $$\langle V\rangle =f_{2}\frac{FD^{2}}{\mu }$$

where *μ* is the viscosity of the fluid and *f*_2_ is a function of dimensionless parameters. At low Reynolds numbers, *f*_2_ depends only on the droplet geometry [31]. 

Figure 2b shows the acoustic pressure simulation results inside the whole droplet. SAWs enter from the right side of the droplet. In the beginning, the acoustic pressure intensity on the right side of the droplet is greater than on the left. The acoustic pressure intensity inside the droplet changes periodically with the AC signal, alternating strength and weakness on the left and right (see Video S1). As far as the acoustic streaming inside the droplet is concerned, because of the bottom surface friction, the flow velocity at the bottom of the droplet was zero and the primary azimuthal rotational flow around the periphery of the droplet generated a secondary bulk circulation flow. The radial inward velocity component near the bottom of the droplet was greater than that in the upper region, and there was a strong swirl motion at the bottom of the droplet [24,32].

## 3. Results

### 3.1. Optimization Analysis of the Location for SAWs to Form Acoustic Swirls in the Droplet 

**Figure 3:**
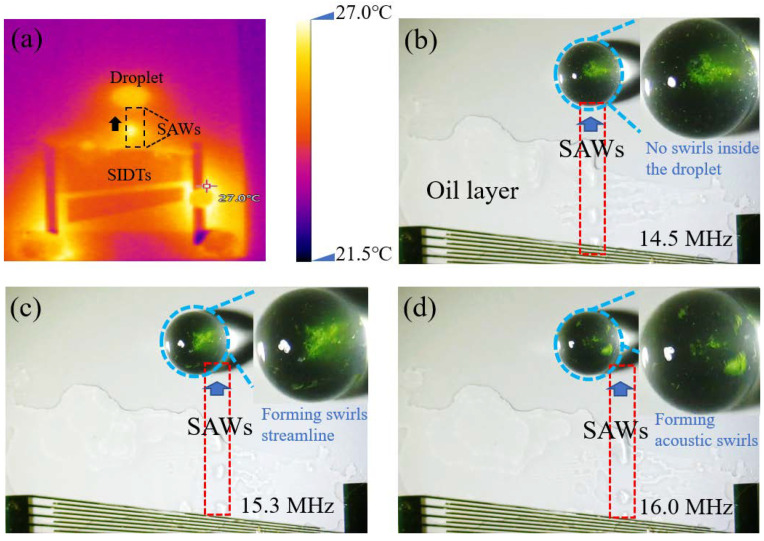
(**a**) Narrow beam of the SAWs photograph taken with a thermal imager. Where the droplet volume is 8 μL. (**b**–**d**) Scattering of SAWs inside the droplet from three different locations after propagation in the oil layer, and SAWs entering the droplet from the center of the droplet (*f* ≈ 14.5 MHz), the center of the right half (*f* ≈ 15.3 MHz), and the edge of the right half (*f* ≈ 16.0 MHz), respectively.

SAWs propagating on the LN surface produced subtle temperature changes. The width of the SAWs was measured with the aid of a thermal imager. It was determined that the SAWs excited by the acoustic microdevices of this design were capable of forming asymmetric effects on a droplet of 8 μL volume, as shown in Figure 3a.

SAWs meeting the droplet in different positions produced different swirl intensities. We designed an experiment to characterize the location of SAW generation. Before the experiment, a layer of silicon polymers was coated on the surface of LN to improve the oleophobic and hydrophobic performance, and to keep the droplet in shape. To better observe the location of SAWs and the acoustic swirl formation, we evenly smeared a Perfluoropolyether (PFPE) lubricating oil layer (Krytox GPL101, DU PONT, Wilmington, DE, USA) between SIDTs and droplets, using a 9 μm PS particle solution to observe the acoustic swirls. Because of the symmetric effect of SAWs on the left and right sides of the droplet, the resonant frequency range of SIDT-excited SAWs was adjusted to the right side of the droplet to simplify the experiment. As a result, the inside of the droplet generated counterclockwise acoustic swirls.

An 8 μL droplet was added to the center of the LN surface using a pipette, and the frequency of SIDTs corresponding to the center of the droplet was *f* ≈ 14.5 MHz (see Figure 3b). When SAWs were excited, it provoked oil beads in the oil layer and the oil beads moved forward in the direction of the propagation of SAWs. SAWs (*f* ≈ 14.5 MHz, *V_PP_* ≈ 37 V) entered from the center of the droplet without creating swirls. The intensity of the swirl formed by the droplet was weak under the action of SAWs with *f* ≈ 15.3 MHz, *V_PP_* ≈ 37 V, and the SAWs located in the center of the right half of the droplet (see Figure 3c). When the excitation frequency was 16.0 MHz, *V_PP_* ≈ 37 V, the SAWs entered the inside of the droplet along the edge of the droplet (see Figure 3d), the particles moved along the swirl streamline, and the acoustic swirls were more obviously observed (see Video S2).

### 3.2. The 9 μm PS Particles in Droplet Driven by SAWs to Form Acoustic Swirls

**Figure 4:**
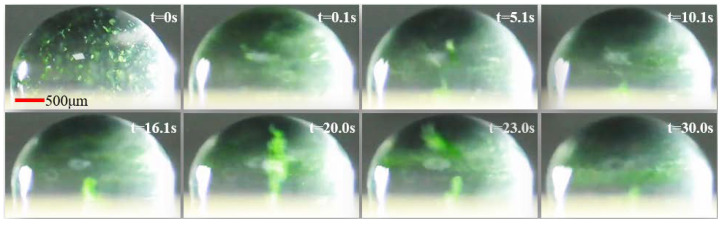
The photographs of the 9 μm PS particles driven by SAWs to form acoustic swirls in 8 μL droplet.

According to the experimental results, the apparent acoustic swirls generated by SAWs from the droplet boundary into the droplet interior. A droplet with the same volume (8 μL) was placed in the same position as Figure 3b, applying a signal with *f* ≈ 16 MHz, *V_PP_* ≈ 37 V, making SAWs enter from the edge of the droplet. The formation of acoustic swirls by particles inside droplets within 30 s from SAWs-on was recorded under the microscope, as shown in Figure 4 (see Video S3). When the SAWs were turned on, the particles inside the droplet began to form the prototype of acoustic swirls under the action of acoustic streaming at 5.1 s. The particles started to move along the swirl streamline, and due to surface friction, the particle velocity at the bottom is lower. Even though the flow swirled upwards through a secondary circulation central column of the flow, the particles clustered at the center of the column in a conical shape, and the particles at the top rotated at a high speed (see Figure 4, t = 16.1 s~30.0 s).

### 3.3. Testing of the Stirring Abilities of Acoustic Swirls

**Figure 5:**
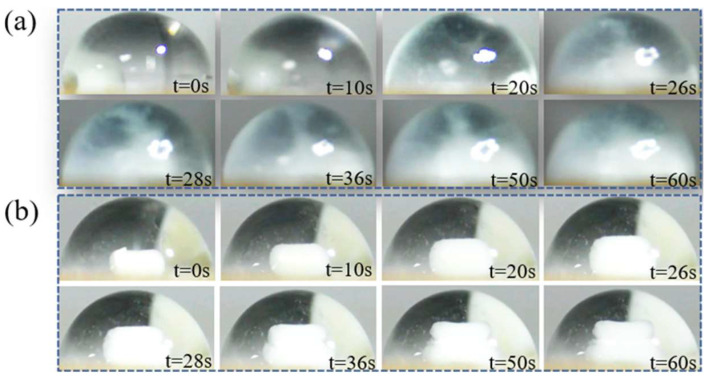
Effect of acoustic swirls on the dissolution of yeast cell powder granules. (**a**) When SAWs are ON, use the acoustic swirls to stir yeast cell powder granules for 60 s. (**b**) When SAWs are OFF, the changes of one yeast cell powder granules in stationary droplets for 60 s (as a control experiment).

To test the stirring abilities of the acoustic swirls, we used the yeast cell powder granules for demonstration. As shown in Figure 5, the yeast cell powder granules were placed on the surface of the LN and then an 8 µL droplet was added from the top of the yeast cell powder granules so that it was completely enveloped by the droplet.

When the SAWs were ON, the yeast cell powder granules started to dissolve and the rate of dissolution became gradually accelerated. The inside of the droplet also became cloudy from its initial clear state. Yeast cell powder granules were fully dissolved and filled the whole droplet interior after 60 s as shown in Figure 5a (see Video S4). Figure 5b depicts the changes in yeast cell powder granules in a stationary droplet at 60 s. When the SAWs were OFF, the yeast cell powder granules only expanded and did not fully integrate with the droplet, and most areas of the droplet still were transparent.

## 4. Discussion

After investigating the strength of the acoustic swirls generated by SAWs coupled into the droplet from different places (see Figure 3b–d), it was found that the thickness of the oil layer had a weakening effect on the energy of SAWs. The thin oil layer was chosen to be closer to the real SAWs, and the experiments showed that the acoustic swirls produced by SAWs coupled into the droplet from the droplet edge were most pronounced during the sweep of SAWs from the middle to the edge of the droplet (14.5 MHz to 16.0 MHz). In the experiments with a droplet containing 9 µm PS particles, the droplet placement was not the same as the droplet placement in Figure 3b, and the resonant frequency needed to be fine-tuned when exploring the excitation frequency required for SAWs to induce acoustic swirls formation. After repeated experiments (more than three times), it was discovered that the formation time of the acoustic swirl induced by the acoustic microdevice in the droplet containing 9 μm PS particles was about 5 s (see Figure 4, t = 5.1 s). However, when stirring yeast cell powder granules, acoustic swirl formation gradually occurred at 26 s (see Figure 5, t = 26 s). Therefore, different particle sizes and fluid types affected the acoustic swirl. G. Destgeer et al. found that the aggregation effect of different particle sizes varied significantly even at the same frequency [27]. The viscosity of the solution influenced the swirl velocity. According to the conclusion of A. Riau et al., the higher the viscosity of the fluid, the lower the swirl velocity inside the fluid [31]. J. A. Garcia-Merino et al. [33] studied distilled water, ethanol, and specific viscous mediums containing nanoparticles and found that the geometry of droplets formed by fluids with different viscosities was slightly different. Therefore, when stirring different types of agitated materials with acoustic swirl, it is necessary to consider not only the size of the agitated material but also the viscosity and the geometry of the fluid. In addition, acoustic swirls have great potential for stirring solids, liquids, etc., in specific application environments. The experimental results of this paper indicate that if the acoustic stirring device is combined with the biosensor, it will assist in the preparation or treatment process of biological samples and may improve the performance of the biosensor. In the future, acoustic swirls can be used in the configuration of fine-concentration solutions, and have important prospects in micro-biological and chemical reactions.

## 5. Conclusions

In this work, we successfully induced acoustic swirls inside μ-droplets. The SAWs propagation pattern and acoustic pressure distribution inside the droplet were simulated in 2D and 3D. We explored that the SAWs enter from different positions of the droplet to obtain different acoustic swirl intensities, and the acoustic swirls gradually increased during the movement of SAWs from the center to the edge of the droplet. The particles within the droplet gathered at the bottom at the beginning, and as the frequency changed, the particles moved along the swirl streamline. The results indicate that the stronger acoustic swirls were formed by SAWs coupled in from the edge of the droplet at the same power. The tunable acoustic microdevice enabled the generation of SAWs with different frequencies on a single device, which rapidly excited the resonant frequencies of different droplet positions required by the acoustic swirl. With a wide range of operating frequencies, placing droplets at any position of the aperture of SIDT, was compatible with the stirring function, which reduced the difficulty of precise position fixing of droplets by using a traditional device. Acoustic microdevice stirring performed well in different volumetric microliter solutions, bringing the merits of abandoning large batches of solutions. Benefiting from our device, the application of acoustic swirl accelerated the dissolution and diffusion of the stirred material in the solvent, such as yeast cell powder granules. 

## Data Availability

The data that support the findings of this study are available from the corresponding author upon reasonable request.

## Supplementary Materials

The following supporting information can be downloaded at: https://www.mdpi.com/article/10.3390/mi14040837/s1. Video S1: Simulation results of SAWs propagating in the form of Rayleigh angles inside droplets (2D), and SAWs forming swirl-like acoustic pressure inside droplets (3D). Video S2: Changes of acoustic swirls intensity during the sweep of SAWs (characterization by oil) from the center to the edge of the droplet at the same power with frequency changes from 14.5 MHz to 16.0 MHz. Video S3: 9 μm PS particles driven by SAWs to form acoustic swirls inside the droplet. Video S4: Acoustic swirls actuate the stirring and dissolving of yeast cell powder granules in the liquid droplet, compared with no treatment condition in a control experiment.
